# Countermovement jump force-time waveforms in male adolescent footballers with growth-related musculoskeletal pathology

**DOI:** 10.3389/fspor.2026.1887562

**Published:** 2026-08-25

**Authors:** Sam Blanchard, Sinead Holden, Matthew Taberner, Liam Dadd, Sean Cumming, Sean Williams, Steffi Colyer

**Affiliations:** 1 Department of Health, University of Bath, Bath, United Kingdom; 2 Manchester City Football Club, Manchester, United Kingdom; 3 School of Public Health, Physiotherapy and Sports Science, University College Dublin, Dublin, Ireland; 4 Research Institute of Sport and Exercise Sciences, LJMU, Liverpool, United Kingdom

**Keywords:** adolescent athletes, bilateral asymmetry, force-time curve, growth-related injury, Osgood-Schlatter disease, pars interarticularis fracture, spondylolysis

## Abstract

**Introduction:**

Growth-related musculoskeletal pathology is common in adolescent athletes, although associated changes in countermovement jump force-time characteristics remain poorly understood. This exploratory repeated-measures biomechanical analysis examined whether adolescent footballers with current or historical growth-related pathology, including Osgood-Schlatter disease (OSD) and prior pars interarticularis fracture, displayed limb-specific differences in double-leg countermovement jump (DL-CMJ) force-time waveforms not evident using discrete performance metrics alone.

**Methods:**

Five male academy footballers completed 15-20 DL-CMJ testing sessions, representing presentations of developing OSD, resolved OSD, resolved OSD with prior pars fracture, and two pain-free reference athletes. Limb-specific vertical ground reaction force waveforms were time-normalised and analysed using one-dimensional Statistical Parametric Mapping.

**Results:**

Athletes with growth-related pathology displayed phase-specific waveform asymmetries, altered propulsive work distribution, and divergent reversal timing patterns compared with pain-free reference athletes. Force-time differences were observed both during symptomatic periods and in athletes with historical pathology who remained pain-free at the time of testing.

**Discussion:**

These findings suggest that waveform analysis may provide additional insight into movement strategies associated with growth-related musculoskeletal pathology beyond conventional countermovement jump metrics. Further longitudinal investigation in larger cohorts is required to determine the clinical relevance of these observations.

## Introduction

The double-leg countermovement jump (DL-CMJ) is widely used to assess neuromuscular performance in elite athletes (1, 2), and dual-force-plate technology enables routine collection of limb-specific kinetics across all jump phases (3). However, discrete outcome measures such as jump height or peak force provide limited insight into how force is distributed throughout the movement or how injury affects limb-specific loading strategies (4, 5). Findings linking DL-CMJ outputs to injury risk are inconsistent: jump height shows little association with injury (6), prior injury remains a stronger predictor than CMJ metrics (7), and interlimb differences may persist following return to play (8). These inconsistencies may partly reflect the limitations of discrete metrics, which do not capture the temporal structure of force production. Previous research has shown that lower-limb pathology can be associated with altered interlimb force characteristics during bilateral jumping tasks, particularly during the eccentric braking phase (9), supporting the examination of force-time waveforms when exploring movement adaptations associated with growth-related musculoskeletal pathology.

Analysing the full force-time waveform, rather than isolated discrete variables, may provide greater insight into movement strategy and enable identification of temporal changes in force production that may not be reflected in global performance outcomes (10). Previous injury may therefore be associated with altered force-time characteristics despite preserved jump height or overall performance (11). Repeated within-athlete waveform analysis may be particularly useful in identifying individualised adaptations that are diluted or absent in group-level analyses (12, 13). By applying one-dimensional Statistical Parametric Mapping to repeated within-athlete observations, localised waveform deviations can be identified that reflect altered neuromuscular strategies rather than gross performance decrements (14). Evidence from athletes following anterior cruciate ligament reconstruction further supports this approach, with force-time waveform analysis identifying persistent movement alterations not consistently detected using traditional discrete metrics (15).

Osgood-Schlatter disease (OSD) is a common overuse injury in adolescents, with prevalence around 10% in 12–15-year-olds and higher rates in athletic populations due to repetitive loading of the tibial tuberosity (16, 17). Pars fractures are 2–5 times more common in athletes than non-athletes and are associated with substantial time-loss in football (18, 19). A recent study identified a potential association between OSD and pars fractures, proposing that compensatory strategies adopted in response to OSD-related pain may contribute to altered proximal loading patterns (20). This may reflect broader skeletal vulnerability during rapid growth, where increases in muscle mass can precede peak bone mineral content (21, 22), exposing immature bone to higher torques (23). In symptomatic OSD, altered kicking mechanics including reduced movement variability and increased knee extension angular velocity have been observed despite maintained ball speed (24), potentially reflecting compensatory strategies that redistribute load proximally. Similar interlimb compensations have previously been observed following ACL reconstruction (9). Although the relationship between OSD, altered movement strategy, and pars pathology remains speculative, these observations highlight the importance of examining complete force-time waveforms and interlimb asymmetries rather than relying solely on discrete performance metrics.

The aim of this study was to use waveform analysis of DL-CMJ ground reaction forces to examine longitudinal force production adaptations in adolescent athletes with and without OSD and pars fractures across 15–20 weeks of monitoring. We hypothesised that players with existing or historic OSD would demonstrate altered force-time characteristics and interlimb asymmetries compared to asymptomatic reference players, and that the athlete with combined historic OSD and pars fracture would display persistent asymmetry and altered temporal reversal patterns.

## Materials and methods

We conducted an exploratory repeated-measures biomechanical analysis of five adolescent football athletes, including prospective and historic examples of growth-related injuries (GRI). Reference athletes were included to provide contextual interpretation of waveform variability and asymmetry patterns rather than formal inferential comparison. As part of routine monitoring, players completed CMJ testing once per week on the last training session before a competitive match (MD-1). For the five presentations included here, we analysed 15–20 testing sessions per athlete (3 valid DL-CMJs per session), yielding 45–60 jumps per player to be subsequently analysed. Ethical approval was obtained from the University of Bath Biomedical Sciences Research Ethics Committee (ref: 8,612–13,425). Written informed consent was obtained from all parents/guardians prior to testing.

### Participants

Participants were recruited from an English Premier League football academy competing at a Category 1 level. Players from the U13, U14, U16, and U18 squads underwent routine testing and monitoring as part of normal club training and management. From this larger cohort, five athletes were purposively selected for detailed waveform analysis based on GRI status and data completeness. Baseline participant characteristics, including maturity status, growth rates, injury history, and symptom status, are presented in Table 1.

**Table 1:** Baseline characteristics of the five presentations, including maturity status (percentage of predicted adult height), growth rates, injury history, and symptom status at the time of testing.

Individual	Chronological age	Percentage of Predicted Adult Height	Growth rate preceding testing (cm/year)	Growth rate at point of testing (cm/year)	Height (cm)	Body Mass (kg)	Injury History	Symptom status
Presentation 1	13.0	91.2	4.35	6.88	167.2	49.6	Developed OSD during testing window	Pain free for 26 sessions, pain present for 9 sessions
Presentation 2	13.7	86.9	4.78	6.56	157.2	44.8	None	Pain free
Presentation 3	16.7	99.3	1.62	6.08	180.7	64.0	2-year history of OSD	>12 months pain free
Presentation 4	16.5	99.6	0	4.78	176.4	71.0	3-month history of OSD followed by Pars Fracture	>12 months pain free
Presentation 5	17.8	99.8	0	0	185.4	77.0	None	Pain free

Osgood-Schlatter disease (OSD); pars interarticularis fracture (pars fracture).

### Procedure set up

Players were instructed to keep to their normal daily routines. Testing took place within the same time-window on each testing day throughout the pre-season, 0900–1,000 for U14-U18s. The U13 testing group were collected on their arrival from school, which was 1,400 for those on a Full Time Training Model (in-house education provided around training sessions) and between 1,700–1,800 for those on a Hybrid Training Model (that remained in school throughout the entire day).

The players completed a standardised warm-up each day which consisted of a) self-mobilisation using a foam roller and/or resistance band, b) 15 m A-Skips, c) 15 m walking lunges, d) 15 m high knee bounds, e) 15 m Squat jumps, f) single leg isometric bridge 3 × 6 s each leg, g) bridge walk-out x 5 reps, h) 10x Pogo hops, i) 3x sub-maximum CMJ's. Each player conducted the CMJ testing within 5 min of completing the warmup.

A standardised methodology for CMJ was followed (25–27). CMJ's were performed on a dual-force plate system (ForceDecks MAX–VALD Performance, Brisbane, Australia) connected to a Dell Latitude 7,320 laptop computer. Both force plates ran at a sampling frequency of 1,000 Hz. A known weight (15 kg) was placed on the plates prior to testing each morning, with an acceptable weight tolerance of ±0.1 kg. To ensure the plates were level, an inclinometer app was used (Angle Finder, JRSoftWorx). The player stood with one foot on each plate, wearing training shoes provided by the club, with hands on hips and ensured they stayed still for approximately five seconds to record a stable body weight. The force trace was displayed on a large TV screen in front of the athlete to provide visual feedback on a stable trace prior to each jump. The athlete was instructed to keep their hands on their hips for each jump and given the verbal cue “jump as fast and as high as you can, stick your landing”. Once a stable trace had been achieved, the players were counted down to each jump with the phrase “3, 2, 1, Jump!” No further coaching cues were given throughout the test. Using the VALD Hub website (VALD Performance, Australia), a competitive leaderboard was set up for each squad on back-to-back testing days. Players performed three maximal jumps separated by 15 s and a quiet, stable trace was ensured after players readjusted their feet from landing. Any jumps deviating from the standard protocol (e.g., jumpers attempted to “tuck” their legs during the flight phase, double jump/prejump, and did not land on the force plates) were excluded, and another jump was performed to ensure three acceptable trials (26).

### Inclusion and exclusion criteria

Players were included if they were registered with the club, fully available for squad training (U13, U14, U16 and U18 squads), and had at least one year's familiarisation with the DL-CMJ protocol (minimum four testing windows). Players were excluded if a pathology prevented back-to-back testing (as determined by the medical team), if they were unfamiliar with the protocol, or if they were unavailable for the first post off-season testing session. OSD and pars fractures were diagnosed using established clinical criteria and imaging confirmation where appropriate, following procedures described previously (20).

Individuals were selected for this analysis based on the following criteria:

- Presentation 1 “Developing OSD”; Player pain-free at baseline but developed OSD during the study. Comparisons of painful and non-painful sessions within the same athlete were used to examine within-athlete changes in force-time characteristics while reducing the influence of maturational progression. A baseline mean vGRF waveform was created from all pain-free trials (36 DL-CMJs across 12 testing timepoints), and a *Δ*force waveform (pain—baseline) was computed for each painful session (9 DL-CMJs across 3 testing time points).
- Presentation 2 “Pain-Free Reference 1”; Pain-free athlete from the same age group and training environment as Presentation 1. Comparison of *Δ*force waveforms between Presentation 1 and the Pain-Free Reference athlete provided contextual interpretation of whether observed changes during painful sessions differed from typical within-athlete variability in a similarly maturing player.
- Presentation 3 “Resolved OSD”; Athlete with a history of OSD (>12 months prior to testing) who remained pain-free throughout the monitoring period.
- Presentation 4 “Resolved OSD with prior Pars Fracture”; Athlete with a history of OSD and prior pars interarticularis fracture (>12 months before testing) who remained pain-free throughout the monitoring period.
- Presentation 5 “Pain-Free Reference 2”; Pain-free athlete from a similar maturational stage and training environment to Presentations 3 and 4, included to provide contextual interpretation of waveform variability and asymmetry patterns.

### Data collection

Each DL-CMJ recorded bilateral vertical ground reaction force (vGRF) data using a dual-force plate system (ForceDecks MAX-VALD Performance, Brisbane, Australia) sampling at 1,000 Hz. Three valid trials were collected during each testing session and exported for subsequent waveform and discrete variable analysis

### Estimated maturation

Stature, sitting height, and body mass were assessed every 12 weeks following ISAK-recommended procedures. Lower-limb length was estimated by subtracting sitting height from stature. Growth rates (cm/year) were calculated for stature and lower-limb length from the measurement window prior to testing and at the point of testing. Predicted adult height was estimated using the Khamis-Roche method (28), and somatic maturity was expressed as percentage of predicted adult height (%PAH). Players were classified as approaching PHV (<85% PAH), circa-PHV (85%–96%), or post-PHV (>96.1%) (29).

### Data analysis

Raw vertical force data were processed using a custom Matlab (R2025a) script. A fourth-order low-pass Butterworth filter with a 50 Hz cut-off frequency, determined using residual analysis, was applied to all force signals prior to analysis. Body weight was calculated from 1 s of quiet standing. Movement onset was defined as the instant summed vertical force fell >2 SD below body weight for ≥0.1 s, and take-off was defined as the instant summed force fell below 10 N.

For waveform analysis, limb-specific vertical ground reaction force (vGRF) data from movement onset to take-off were time-normalised to 101 points (0%–100% of movement) to enable node-by-node comparison across trials and athletes. For Presentation 1 (Developing OSD) and Presentation 2 (Pain-Free Reference 1), mean baseline waveforms were created from all pain-free trials collected during the monitoring period. *Δ*force waveforms were subsequently calculated for painful sessions relative to the baseline waveform to examine within-athlete changes in force production during symptomatic periods while reducing the influence of baseline inter-athlete differences and maturational progression.

Discrete variables were derived from the non-normalised force data. Relative vertical impulse (m/s) for each limb was calculated by integrating net vertical force (force minus half body weight) and dividing by body mass. Limb-specific reversal point (LRP) was defined as the percentage of movement at which relative impulse transitioned from negative to positive, representing the shift from net braking to net propulsion. Whole-body bottom of countermovement (BCM) was identified as the point where centre-of-mass velocity transitioned from negative to positive. Early ascent impulse rate (m/s·s^−1^) was calculated as the average rate of increase in relative impulse during the first 100 ms of the concentric phase. Limb-specific power was calculated as force multiplied by centre-of-mass velocity, and negative and positive work (J) were determined by integrating power across the eccentric and concentric phases, respectively. Full computational definitions are provided in Supplementary File S1.

Waveform analyses were conducted using one-dimensional Statistical Parametric Mapping (spm1d) (Pataky, 2010) on the 101 time-normalised nodes. Random-field-theory-based thresholds were applied at *α* = 0.05 to identify supra-threshold clusters representing time-localised waveform differences. Effect sizes were expressed as one-dimensional Cohen's d curves and interpreted using established thresholds, with 1.2, 2.0, and 4.0 representing large, very large, and extremely large effects, respectively Hopkins et al. (31). Full statistical procedures and discrete-variable analyses are provided in Supplementary File S2.

### Statistical analysis

Waveform analyses used one-dimensional Statistical Parametric Mapping (spm1d) (30) on 101 time-normalised nodes, with random-field-theory-based thresholds at *α* = 0.05 to identify supra-threshold clusters indicating time-localised differences. Effect sizes were expressed as 1D Cohen's d curves and interpreted using established thresholds, with 1.2, 2.0 and 4.0 denoting large, very large and extremely large effects, respectively (31); full computational details and discrete-variable analyses are provided in Supplementary File S2.

## Results

### Participant characteristics

Baseline participant characteristics are summarised in Table 1.

#### Presentation 1 and presentation 2: developing OSD and pain-free reference 1

**Figure 1:**
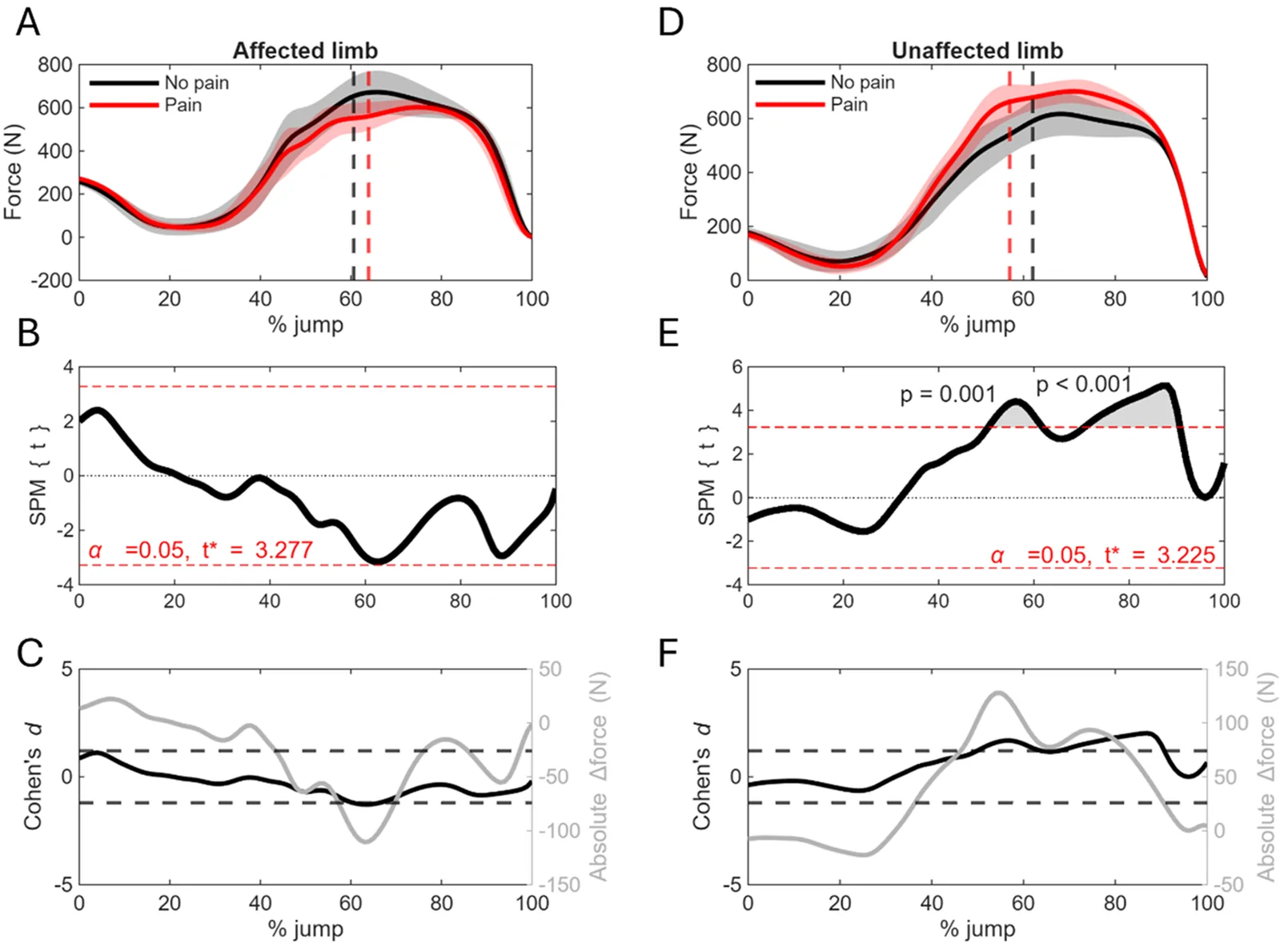
Within-athlete changes in limb loading with the onset of OSD pain (presentation One). Force production of the affected and unaffected limbs in Case One before and after onset of pain. Panels show **(A,D)** mean ± SD force-time waveforms for pain-free and painful conditions (dotted vertical lines = limb-specific reversal points, LRP); **(B,E)** SPM{t} curves with supra-threshold clusters (grey) indicating time-points where force differed significantly between conditions; and **(C,F)** Cohen's d effect-size waveforms and *Δ*force illustrating the magnitude and direction of differences, with the dashed horizontal line marking a large effect size (>1.2).

In the Developing OSD presentation, no suprathreshold clusters were detected in the affected limb when comparing pain-free and painful trials (Figure 1B), although the effect size curve approached large magnitude during late descent (≈58%–70% of the jump; Figure 1C). During pain, the affected limb showed lower early ascent impulse rate (*p* = 0.036, ES = −1.3) and reduced positive work (*p* = 0.019, ES = −1.4) compared with pain-free trials. Limb-specific discrete variables are reported in Table 2.

**Table 2:** Limb-specific countermovement jump discrete variables describing ’speed out of the bottom’ and propulsive capacity for each presentation.

Presentation	Condition	Limb	Eccentric Velocity (m/s)	Rate of Velocity Change Ascent (m/s^2^)	Negative Work (J)	Positive Work (J)
Presentation 1	No Pain (Baseline)	Affected	−0.58	8.33	−26.04	89.65
Presentation 1	No Pain (Baseline)	Unaffected	−0.56	6.83	−28.88	77.30
Presentation 1	With Pain	Affected	−0.66	5.29	−28.46	54.71
Presentation 1	With Pain	Unaffected	−0.60	9.59	−26.93	116.12
Presentation 2	Pain-Free Reference 1	Left	−0.65	5.22	−38.80	92.98
Presentation 2	Pain-Free Reference 1	Right	−0.57	6.69	−30.11	113.20
Presentation 3	Resolved OSD	Affected	−0.66	6.35	−39.61	77.59
Presentation 3	Resolved OSD	Unaffected	−0.88	7.91	−58.20	154.00
Presentation 4	Resolved OSD + Pars	Affected	−0.75	5.34	−61.10	153.76
Presentation 4	Resolved OSD + Pars	Unaffected	−0.71	9.50	−54.00	247.34
Presentation 5	Pain-Free Reference 2	Left	−0.66	8.04	−46.02	183.69
Presentation 5	Pain-Free Reference 2	Right	−0.67	9.18	−48.18	195.23

Eccentric velocity reflects maximum vertical drop speed into the bottom of the countermovement, the rate of velocity change in ascent represents the average gradient of limb-specific relative impulse over the first 100 ms of the ascending phase (indicator of speed out of the bottom), and positive work is derived by numerically integrating power (force × relative impulse) during the propulsive phase.

**Figure 2:**
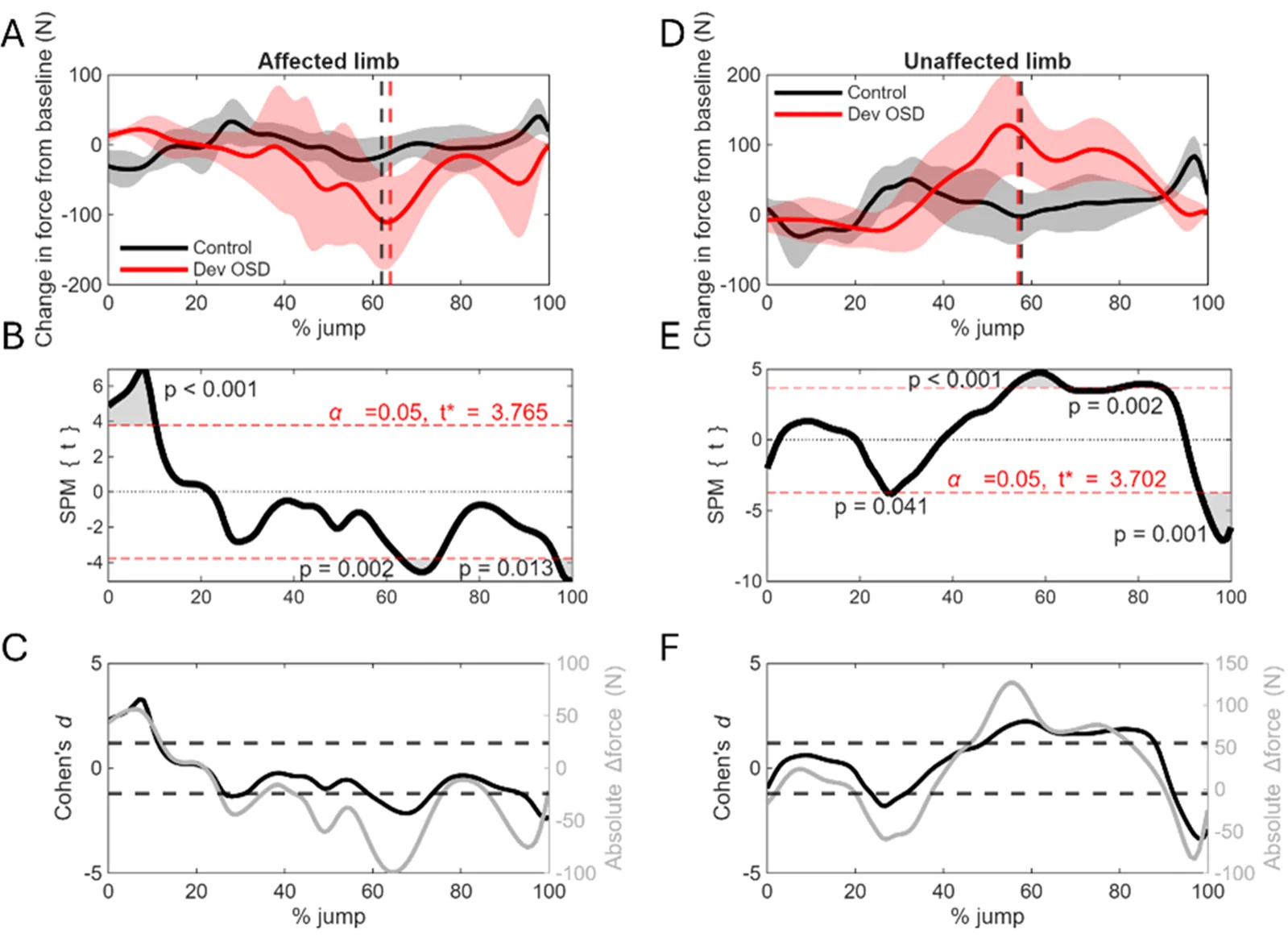
Pain-related changes from baseline in presentation One compared with a pain-free reference (presentation Two). Changes in force production from baseline in Presentation One (Developing OSD) relative to Presentation Two (Pain-Free Reference 1). Panels show **(A,D)** mean ± SD *Δ*force waveforms (dotted vertical lines = LRP); **(B,E)** SPM{t} outputs with grey regions indicating significant between-player differences; and **(C,F)** Cohen's d effect-size waveforms depicting the magnitude and direction of between-player differences, with the dashed horizontal line indicating a large effect size (>1.2).

In contrast, the unaffected limb produced greater vertical force during pain between 51%–61% and 71%–90% of the jump (*p* = 0.001 and *p* < 0.001; mean *d* = 1.3–1.8; Figure 2B). Consistent with this, early ascent impulse rate and positive work were higher during pain (*p* = 0.027 and *p* = 0.013; ES = 1.4 and 1.5), with positive work more than double that of the affected limb (116.1 ± 29.2 J vs. 54.7 ± 31.9 J).

Compared with Pain-Free Reference 1, the affected limb of the Developing OSD example showed significant *Δ*force differences during early descent (0%–10% of the jump; *p* < 0.001, d > 2.8), where *Δ*force was negative (Figure 2C). Additional clusters occurred at 64%–71% (*p* = 0.002) and 97%–100% (*p* = 0.013), with very large effect sizes (≈ −2.0).

Whole-body BCM occurred slightly earlier during pain (59.7% vs. 61.2%). Limb-specific LRPs diverged, with the affected limb occurring later (60.6% to 62.0%) and the unaffected limb earlier (63.9% to 57.2%). This reversed the baseline pattern, with the unaffected limb initiating propulsion 4.8% earlier during pain.

#### Presentation 3: resolved OSD

**Figure 3:**
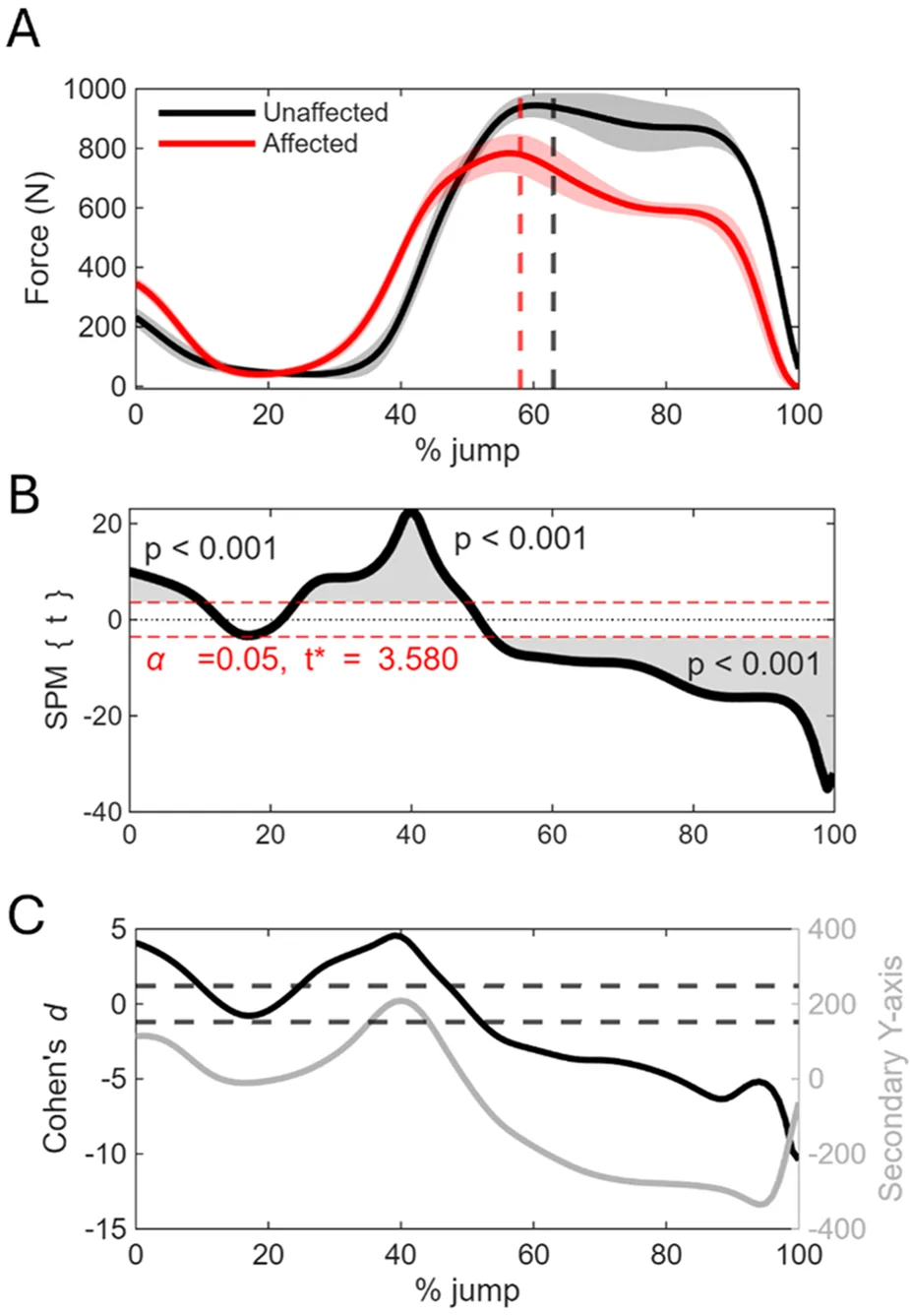
Bilateral force-time asymmetries in a player with resolved OSD (presentation three). Bilateral force production in Presentation Three (Resolved OSD). Panels show **(A)** mean ± SD force-time waveforms for affected and unaffected limbs (dotted vertical lines = LRP); **(B)** SPM{t} curve with grey regions indicating significant bilateral differences; and **(C)** Cohen's d effect-size waveform quantifying asymmetry magnitude, with the dashed horizontal line representing a large effect size (>1.2).

In the Resolved OSD example, the unaffected limb produced greater force than the previously affected limb across most of the movement (Figure 3). Significant bilateral differences occurred during initial unloading (0%–10%) and the eccentric phase (24%–47%) (*p* < 0.001), and throughout late eccentric and propulsive phases (52%–100%; *p* < 0.001). Effect sizes were extremely large at jump initiation (*d* = 4.1) and around 40% of the jump (*d* = 4.6) and remained very large during propulsion (60%–100%; d ≈ −4.8).

The affected limb reversed from eccentric to concentric action earlier (LRP = 58%) than the unaffected limb (LRP = 63%). The unaffected limb therefore produced greater negative work (−58.2 J vs. −39.6 J) and almost double the positive work (≈154 J vs. 78 J).

#### Presentation 4: resolved OSD with prior pars fracture

**Figure 4:**
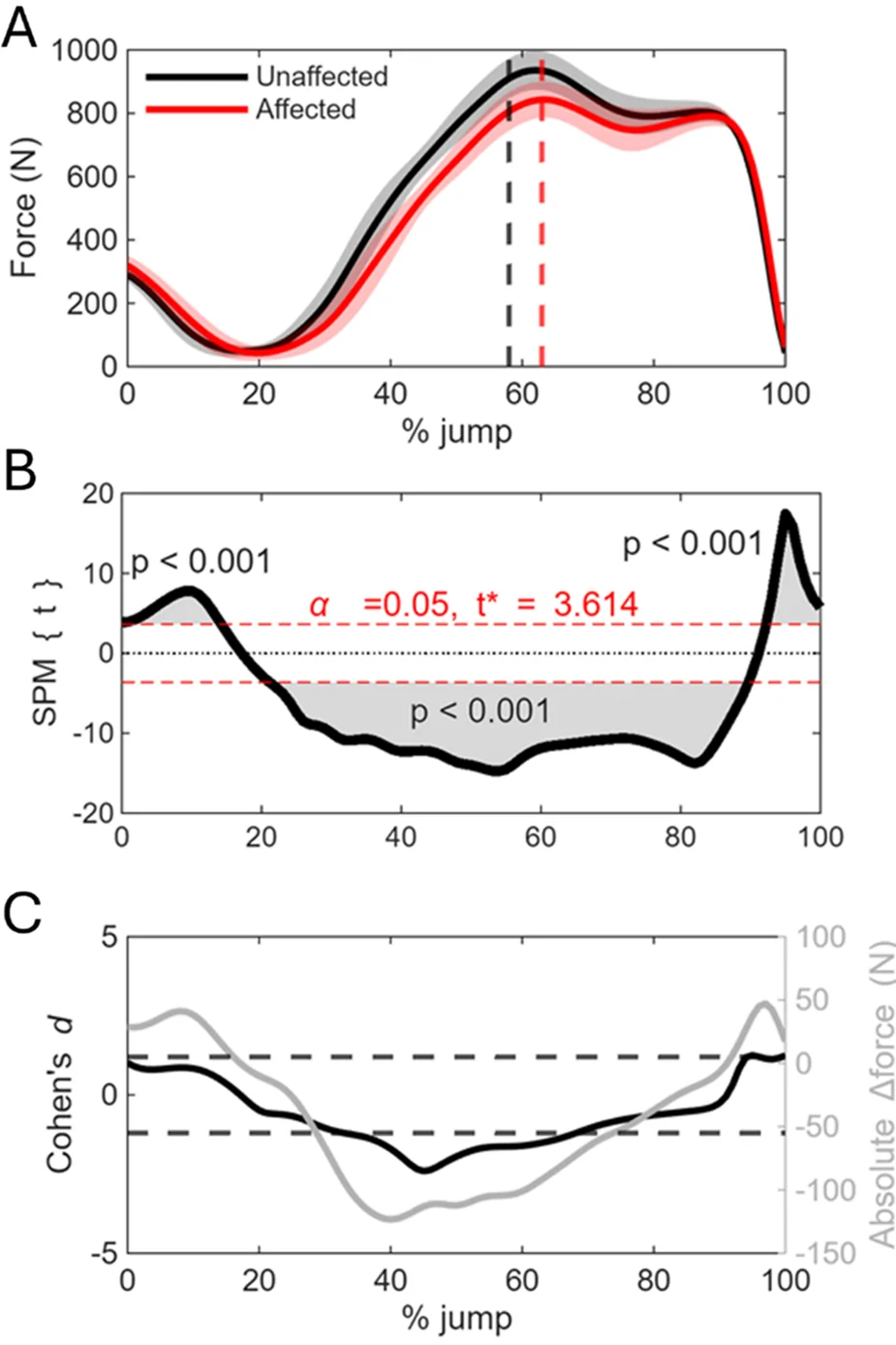
Bilateral force-time strategy in a player with resolved OSD and prior pars fracture (presentation four). Bilateral force production in Presentation Four (Resolved OSD with prior pars fracture). Panels show **(A)** mean ± SD waveforms for previously affected and unaffected limbs (dotted vertical lines = LRP); **(B)** SPM{t} curve highlighting significant bilateral differences (grey regions); and **(C)** Cohen's d effect-size waveform illustrating the magnitude and direction of asymmetry, with the dashed horizontal line marking a large effect size (>1.2).

In the athlete with resolved OSD and prior pars fracture, a distinct bilateral pattern was observed (Figure 4). The previously affected limb produced greater force early in the movement (0%–14%; *p* < 0.001), but lower forces from 22%–89% (*p* < 0.001), with large effect sizes between 31%–72% (*d* = −1.6, peaking at −2.4 at 46%). In the final phase (93%–100%), the previously affected limb again produced greater force.

The historically affected limb reversed from braking to propulsion later than the unaffected limb (LRP = 62% vs. 56%), produced more negative work (−61.1 vs. −54.0 J), and less positive work (≈154 vs. 247 J).

#### Presentation 5: pain-free reference 2

**Figure 5:**
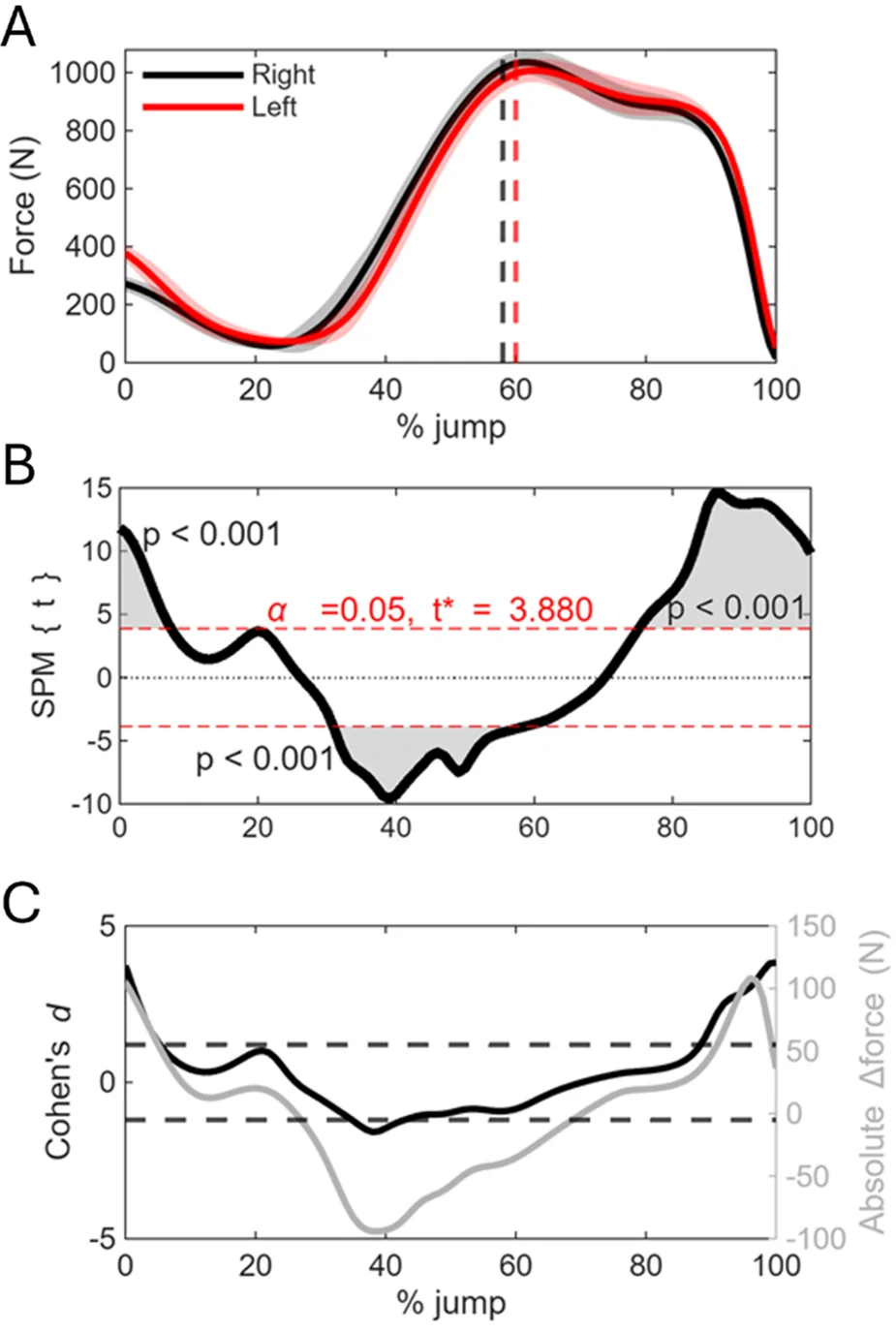
Bilateral force-time characteristics in a pain-free reference (presentation five). Bilateral force production for Presentation Five (Pain-Free Reference 2). Panels show **(A)** mean ± SD waveforms for left and right limbs (dotted vertical lines = LRP); **(B)** SPM{t} curve indicating significant between-limb differences (grey regions); and **(C)** Cohen's d effect-size waveform describing asymmetry magnitude, with the dashed horizontal line denoting a large effect size (>1.2).

In the maturity-matched Pain-Free Reference with no history of OSD or pars fracture, bilateral vGRF differences were smaller and more balanced (Figure 5). Significant asymmetries occurred at 0%–7%, 31%–58%, and 76%–100% of the jump (all *p* < 0.001). Effect sizes were very large at the start and end of the movement (mean *d* = 2.2 and 1.5; left > right), whereas the right limb produced greater force during 31%–58% (mean *d* = −1.1).

Timing and work were near symmetric. LRPs occurred at 60% and 59% for the left and right limbs, respectively, and positive work was similar (≈184 vs. 195 J).

#### Bilateral asymmetry magnitude across presentations

**Figure 6:**
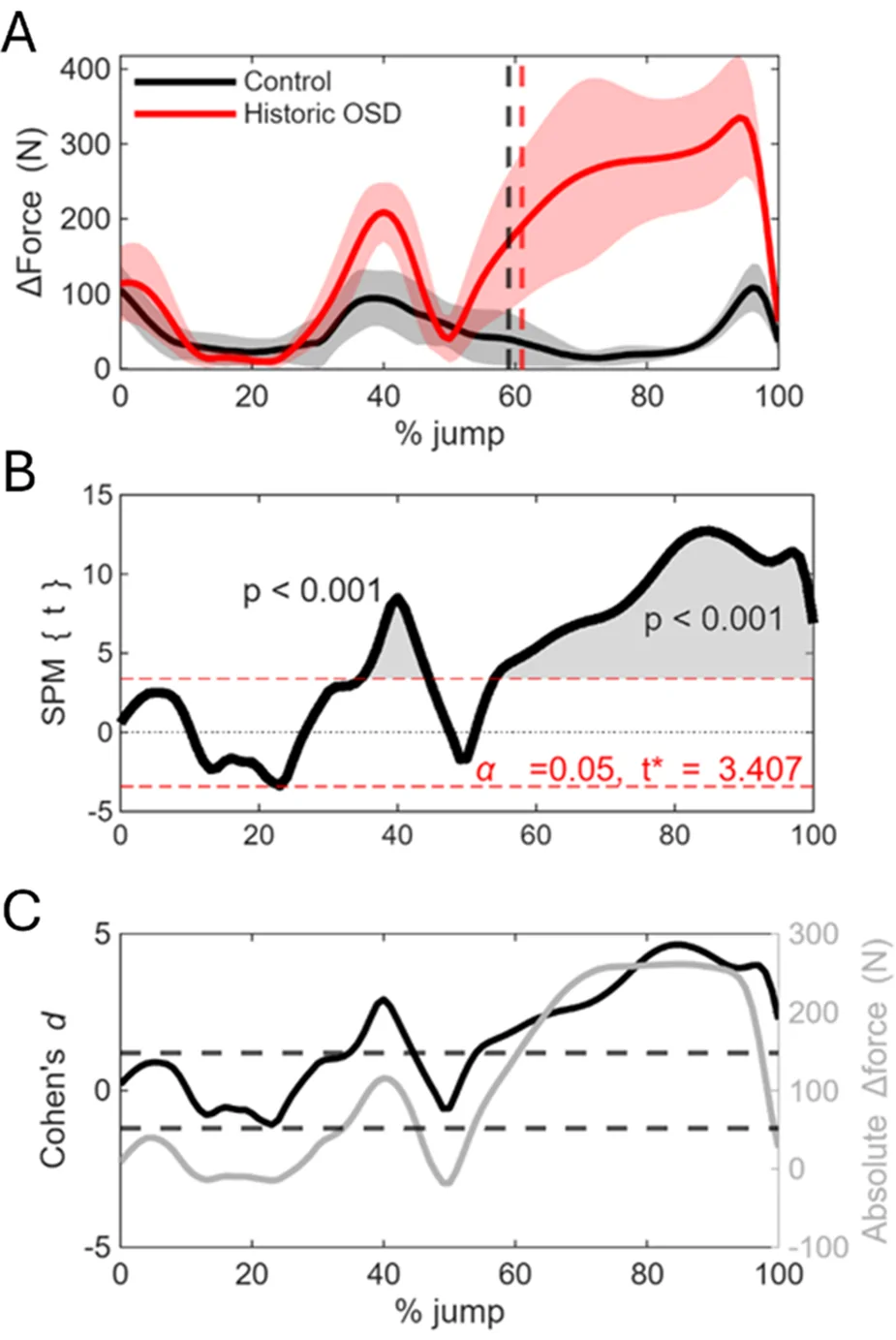
Magnitude of bilateral asymmetry in resolved OSD (presentation three) vs. Pain-Free Reference 2 (Presentation Five). Between-player bilateral asymmetry (*Δ*force) comparison between Presentation Three (Resolved OSD) and Presentation Five (Pain-Free Reference 2). Panels show **(A)** mean ± SD *Δ*force waveforms; **(B)** SPM{t} curve with grey regions indicating significant between-player differences in asymmetry; and **(C)** Cohen's d effect-size waveform, with the dashed horizontal line representing a large effect size (>1.2).

**Figure 7:**
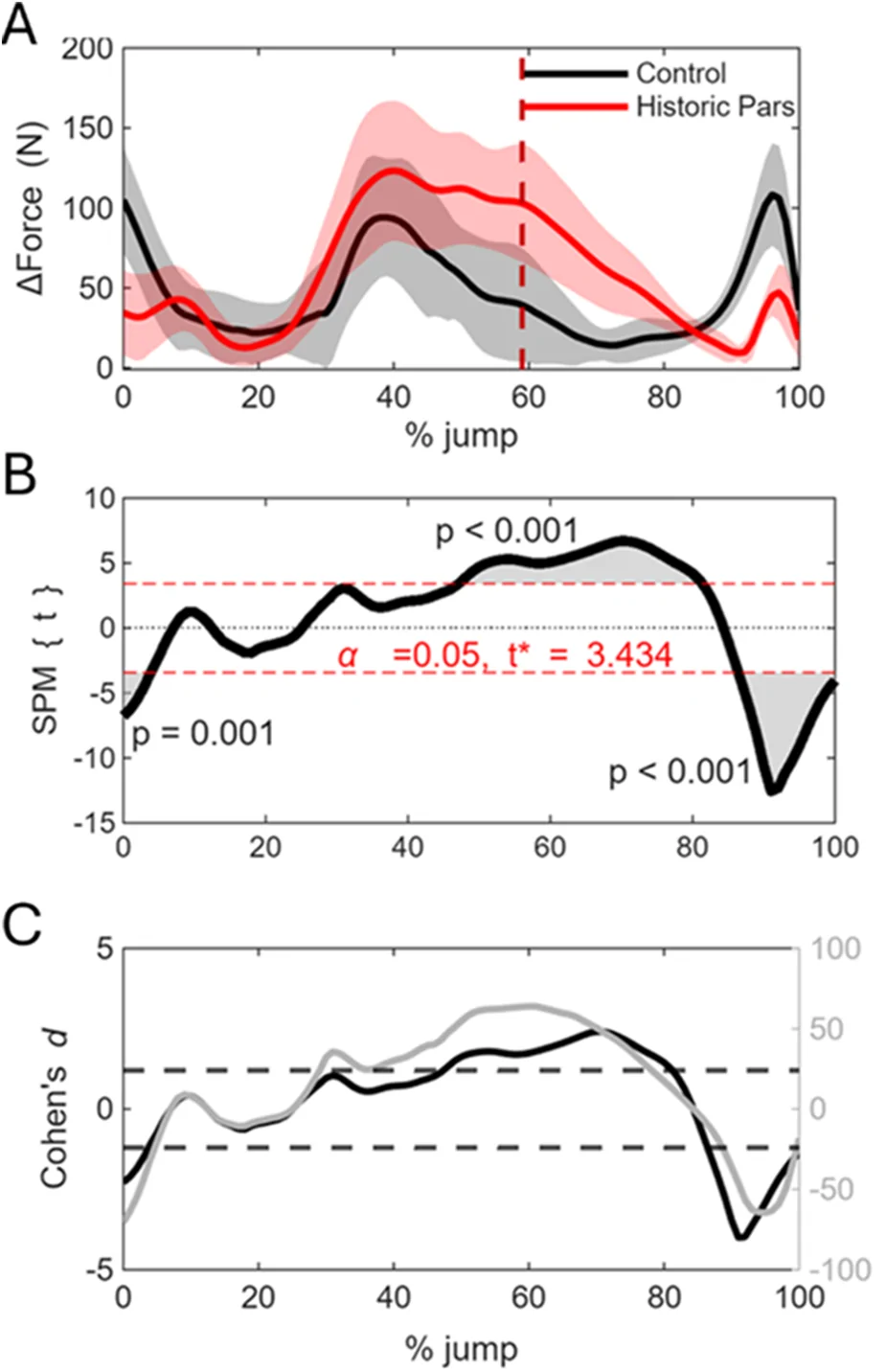
Magnitude of bilateral asymmetry in resolved OSD + pars (presentation four) vs. Pain-Free Reference 2 (Presentation Five). Between-player bilateral asymmetry (*Δ*force) comparison between Presentation Four (Resolved OSD + pars fracture) and Presentation Five (Pain-Free Reference 2). Panels show **(A)** mean ± SD *Δ*force waveforms; **(B)** SPM{t} curve with grey regions indicating significant between-player differences; and **(C)** Cohen's d effect-size waveform, with the dashed horizontal line marking a large effect size (>1.2).

To compare the magnitude of bilateral asymmetry between presentations, absolute *Δ*force (difference between limbs) waveforms were computed for the Resolved OSD example and the Resolved OSD with prior Pars example and contrasted with Pain-Free Reference 2 (Figures 6, 7). Compared with Pain-Free Reference 2, the Resolved OSD example showed substantially greater asymmetry during initial eccentric loading (first rise above body weight) and throughout the propulsive phase, with significant supra-threshold clusters and large effect sizes (Figure 6).

In the Resolved OSD with prior Pars example, asymmetry was greatest in late descent and propulsion (≈48%–81% of the jump), again exceeding that seen in Pain-Free Reference 2 (Figure 7). Although the between-presentation difference in asymmetry magnitude was smaller than that observed for Resolved OSD vs. the reference athlete, effect sizes remained large (mean Cohen's *d* = 1.9), indicating that both historic examples displayed more pronounced and sustained asymmetries than the uninjured reference.

## Discussion

Across 15–20 weeks of monitoring, the GRF waveform analyses revealed two consistent signatures associated with GRIs: (1) magnitude of force asymmetries, with the (previously) affected limb producing lower vertical force through substantial portions of the eccentric and propulsive phases (large to very large effect sizes and sustained supra-threshold SPM clusters); and (2) temporal desynchronisation, reflected in systematic shifts in whole-body bottom of countermovement timing and limb-specific reversal points.

Our original hypothesis proposed that players with existing or resolved OSD would demonstrate reduced eccentric loading through the affected limb, consistent with a pain-driven protective strategy. This was partially supported. The Developing OSD and Resolved OSD presentations both demonstrated a repeatable pattern consistent with what may be described as hesitant eccentric loading with earlier unloading and reduced contribution through the affected limb. However, this pattern was not universal across all presentations. The Resolved OSD with prior Pars Fracture example, having undergone targeted rehabilitation, exhibited a different eccentric loading pattern characterised by a deeper countermovement, greater negative work and a later reversal. Importantly, this occurred alongside a persistent and marked deficit in force and positive work production, indicating that changes in timing or pattern do not reflect restoration of force capacity, with pain also associated with reduced eccentric loading.

The adaptations observed across presentations may be interpreted within a broader framework linking pain, motor behaviour, and movement variability. Motor adaptation theory proposes that pain may be associated with short-term protective changes in movement coordination (32), which could influence how force is distributed during dynamic tasks. When repeated across high-volume tasks such as decelerations and changes of direction, such movement patterns may become more consistently expressed over time. Dynamic systems theory further highlights that movement variability emerges from interacting neuromuscular, mechanical, and perceptual constraints and that both excessive rigidity and excessive variability may increase injury risk (33, 34). Reduced confidence or capacity to tolerate eccentric knee load narrows the available movement solutions, prompting compensatory strategies such as increased reliance on the contralateral limb or greater contribution from proximal segments.

Across all three injury presentations, variations of this shared adaptation were evident through altered force production, temporal reversal patterns, and differences in positive and negative work between limbs. In the Developing OSD presentation, pain was associated with reduced positive work and lower early ascent impulse rate in the affected limb, alongside increased force production and positive work in the unaffected limb during the propulsive phase. Divergent limb-specific reversal points were also observed, with the unaffected limb transitioning into propulsion earlier during painful trials. In the Resolved OSD presentation, the previously affected limb demonstrated earlier reversal timing but persistently lower force production and positive work throughout the eccentric and propulsive phases. In the Resolved OSD with prior Pars presentation, the affected limb exhibited greater negative work and a later reversal point, consistent with a deeper countermovement strategy; however, this did not translate into greater propulsive output, with positive work and force production remaining lower than the unaffected limb across much of the movement cycle. Together, these findings indicate that GRI presentations were associated with altered force magnitude, work distribution, and temporal movement strategies, and that these adaptations may persist beyond symptom resolution. Repeated across the thousands of braking and propulsive actions inherent in football, such asymmetrical loading patterns may contribute to altered stress distribution elsewhere within the kinetic chain. This is consistent with evidence that players with a history of OSD may have a higher risk of pars fracture (20) and that GRIs follow a distal-to-proximal pattern (35).

High-level football requires frequent high-intensity decelerations (36), and players with OSD in the current study demonstrated altered eccentric loading and propulsive force asymmetries. Symptomatic presentations in the current study demonstrated reduced eccentric loading and altered temporal strategies consistent with protective unloading behaviours previously observed in symptomatic OSD. Both resolved presentations demonstrated persistent asymmetries and reduced propulsive output, consistent with reports of prolonged strength deficits following symptom resolution (37). In contrast, ACL literature describes compensations developing from chronic extensor weakness and increased reliance on proximal musculature (38). Together, these findings raise the possibility that pain-related inhibition may, if persistent, be associated with more entrenched strength-based compensations.

Movement variability is now recognised as a functional strategy enabling adaptable motor responses rather than simply reflecting noise (33, 34). The Pain-Free Reference 2, which showed smaller and more balanced asymmetries without the rigid loading patterns seen in the injury examples, is consistent with this view. Following ACL injury, dynamic tasks become more rigid and predictable, and successful rehabilitation is associated with the re-emergence of controlled variability (39). The absence of entrenched compensatory patterns in the Pain-Free Reference may therefore reflect a more adaptable coordination strategy.

Comparable compensatory strategies are seen beyond the knee. Individuals with low back pain exhibit increased trunk stiffness and reduced ability to dampen movement (40), which may parallel the persistent asymmetries and altered temporal force-production strategies observed in the historic GRI presentations. From a complex systems perspective (41), these adaptations emerge from interactions between pain history, tissue capacity, training load, maturation, and behavioural regulation, and GRI should not be viewed as a single determinant of future injury. Nevertheless, the consistent alterations in eccentric strategy and interlimb timing across all three injury examples may highlight a clinically meaningful pattern. The Resolved OSD with prior Pars presentation is particularly illustrative: although the eccentric loading pattern differed following targeted rehabilitation, substantial concentric asymmetries remained, suggesting that temporal force-production adaptations may persist despite partial recovery of force magnitude. Although causality cannot be inferred from this exploratory analysis, the shared force-time adaptations observed here may provide mechanistic insight into this proposed distal-to-proximal progression that warrants further investigation.

To our knowledge, this is the first study to characterise lower-limb GRF waveforms longitudinally across both symptomatic and historic OSD, as well as combined OSD–pars pathology, in elite youth footballers. The integration of SPM-based waveform analysis with repeated weekly testing provides a uniquely sensitive method of detecting subtle, persistent alterations in force magnitude and timing that are not captured by discrete metrics or cross-sectional designs. This approach offers new insight into how GRI shape movement strategies over time and establishes a foundation for future work combining GRF with kinematic and kinetic modelling.

### Limitations

This was a small observational analysis that only utilised kinetic data. We observed global displacement, velocity, impulse and power; however, the absence of kinematic data means we were unable to discuss how that movement is distributed. Future research incorporating joint-specific kinematic analysis (e.g., trunk, hip, knee, and ankle angles) and combining the ground reaction force and kinematic data to provide joint kinetics would be critical to confirm whether compensatory strategies are being employed. In some regions, such as 50%–78% of the jump in the Developing OSD presentation, large effect sizes were accompanied by borderline statistical significance. This suggests a potentially meaningful biomechanical difference, but statistical certainty was limited by the small sample size. Finally, our analysis did not consider the landing phase of the jump, where asymmetries in the presence of injury have been shown to be as high as 57% (42).

Some statistically significant clusters in the final 5%–10% of the jump likely reflect the natural convergence of vertical force toward zero at take-off, which reduces variability and inflates the SPM t-statistic; these late-phase differences should therefore be interpreted cautiously.

## Conclusions

The present exploratory analysis identified distinct force-time waveform characteristics across adolescent football athletes with different presentations of GRI. Limb-specific asymmetry and temporal desynchronisation patterns complemented previous findings that prior injury history may be associated with altered force-time characteristics despite similar global performance outcomes (8, 15). Consistent with waveform-based approaches used in sprint kinetics (4), the current findings suggest that phase-specific waveform analysis may provide additional insight into force production strategies not readily identified using discrete CMJ metrics alone. Notably, force-time differences were observed both during symptomatic periods and in athletes with historical pathology who remained pain-free at the time of testing, although the clinical significance of these observations remains unclear. Future research incorporating larger longitudinal cohorts, combined kinematic-kinetic modelling, and assessment of landing-phase mechanics is required to determine whether these waveform characteristics are consistently associated with GRI during adolescence.

This exploratory retrospective waveform analysis identified distinct force-time characteristics across adolescent football athletes with different presentations of GRI. Temporal waveform analysis appeared sensitive to phase-specific loading differences not consistently reflected by global CMJ performance metrics. These findings support further investigation into whether longitudinal waveform monitoring may provide additional insight into movement strategies associated with GRI during adolescence.

## Data Availability

The datasets presented in this article are not readily available because the data set is property of the football club and under a strict legal agreement between the club and university; we are not able to share the raw data. Requests to access the datasets should be directed to sjb271@bath.ac.uk.
