# Supplementary material for: Countermovement jump force-time waveforms in male adolescent footballers with growth-related musculoskeletal pathology
Source: Front Sports Act Living. 2026 Aug 25;8:1887562. doi: 10.3389/fspor.2026.1887562 (PMC13552125; doi:10.3389/fspor.2026.1887562)
Supplement: Supplementary file 1 [file Supplementaryfile1.docx]

# Supplementary file

## S1. Full computational data analysis

The raw vertical force data were analysed in a custom-written Matlab (R2025a) script, which first applied a fourth order low-pass Butterworth filter (cut-off frequency: 50 Hz, based on residual analysis). The vertical force from the two plates (under each foot) were then summed before a mean vertical force was computed across a one second period of quiet standing and considered to represent the player’s body weight. The onset of movement was defined as the instant where force fell more than 2 standard deviations (SD) (measured across the first second of data) below body weight and remained below this threshold for 100 frames (0.1 s). Take-off was defined as the instant the summed vertical force fell below 10 N. The force data (for each limb) from the onset of movement to take-off were then temporally-normalised to 101 points (nodes, equating to 0-100% of the jump) using spline interpolation.

This temporal normalisation accounted for variation in movement duration both within and between athletes, enabling direct node-by-node comparison across trials and cases.For Presentation One (developing OSD) and Presentation Two (Pain-Free Reference), a mean force trace was computed across all time points where the developing OSD example did not experience pain (26 DL-CMJs). A Δforce from baseline was then computed for all testing instances that occurred during the period when Presentation One was experiencing pain (9 DL-CMJs). This was to overcome the issue that these players exhibited very different force profiles at baseline and to isolate the effect of developing OSD on the force production from any expected maturational changes. A number of discrete outcome variables were also computed from the non-normalised vertical force data. Specifically, the relative vertical impulse (m/s) produced by each limb was computed by numerically integrating the net vertical force data (force minus half of body weight) and dividing by body mass. The minimum relative impulse (maximum change in momentum in descending phase) attributed to each limb was then extracted. The velocity of the centre of mass was also computed from the summed vertical force (across limbs) by numerically integrating the net vertical force. The whole-body bottom of the countermovement (BCM) was then extracted by finding the instant where centre of mass velocity transitioned from negative to positive (rising x-axis crossing in vertical velocity). For each limb separately, the point where relative impulse changed from negative to positive was also extracted, which reflects the point where this limb starts to work propulsively (assuming that body weight is distributed equally, which cannot be directly quantified from these data) and reported as limb-specific reversal point (LRP). As an indicator of speed out of the bottom of the countermovement, the average rate (gradient) of the increase in relative impulse (momentum change) across the first 100 ms of the ascending phase was also calculated. The power produced by each limb was calculated by multiplying the force with the relative impulse (m/s), and the negative and positive work performed across the jump was then computed by numerically integrating the power data for each limb.

## S2: Statistical Parametric Mapping (SPM) framework

All waveform analyses were conducted using one‑dimensional SPM implemented in spm1d (Pataky, 2010). For each comparison, a parametric test statistic (SPM{t}) was computed at each of the 101 nodes along the waveform. Random field theory was used to derive a critical threshold for the SPM{t} field at α = 0.05, accounting for the smoothness and temporal correlation structure of the data. Regions where the SPM{t} curve exceeded this threshold were considered supra‑threshold clusters and interpreted as time‑localised differences between conditions or limbs. For each cluster, the probability of observing a region of equal or greater extent under the null hypothesis was calculated using the SPM framework.

## Effect size calculation

For all waveform tests, a one‑dimensional Cohen’s d curve was computed as mean difference divided by the pooled standard deviation at each node. Effect sizes were interpreted using thresholds of 1.2, 2.0 and 4.0 for large, very large and extremely large effects, respectively (Hopkins et al., 2009). Discrete outcomes were analysed using conventional zero‑dimensional Cohen’s d with the same magnitude thresholds.

## Discrete outcome analysis

Discrete metrics (relative impulse, limb‑specific reversal point, early ascent impulse rate, and negative/positive work) were derived from the non‑normalised vGRF data for each limb. Within‑case bilateral differences were assessed using paired t‑tests, and between‑presentation comparisons were made using independent t‑tests where appropriate. These tests were aligned with the corresponding waveform contrasts but treated as conventional 0D analyses.

**Supplementary Table 1. Summary of one‑dimensional Statistical Parametric Mapping (SPM) tests conducted for each case and contrast:** The table specifies, for each comparison, the waveform outcome analysed (raw limb‑specific vGRF or derived asymmetry waveforms), the contrast of interest (within‑case limb comparison or between‑case condition comparison), and the corresponding SPM test type (paired or two‑sample t‑test). Discrete (0D) analyses of limb‑specific variables followed the same comparison structure and are described in Supplementary file S2

| **Case / comparison** | **Outcome type** | **Contrast description** | **Test type** | **Limbs analysed** |
| --- | --- | --- | --- | --- |
| Presentation 1: Developing OSD – pain‑free vs pain | Waveform vGRF | Within‑presentation condition effect (no pain vs pain) | 1D two‑sample SPM t‑test | Affected, unaffected |
| Presentation 1 vs Presentation 2: Developing OSD vs Pain-Free Reference 1 | Waveform Δforce | Between‑presentation condition effect on limb‑specific Δforce | 1D two‑sample SPM t‑test | Same limb in each presentation |
| Presentation 3: Resolved OSD | Waveform vGRF | Within‑presentation bilateral difference (previously affected vs other limb) | 1D paired SPM t‑test | Both limbs |
| Presentation 4: Resolved OSD + pars fracture | Waveform vGRF | Within‑presentation bilateral difference | 1D paired SPM t‑test | Both limbs |
| Presentation 5: Pain-Free Reference 2 | Waveform vGRF | Within‑presentation bilateral difference | 1D paired SPM t‑test | Both limbs |
| Presentation 3 vs Presentation 5 | Waveform asymmetry | Between‑presentation difference in absolute bilateral asymmetry | 1D two‑sample SPM t‑test | Asymmetry waveform |
| Presentation 4 vs Presentation 5 | Waveform asymmetry | Between‑presentation difference in absolute bilateral asymmetry | 1D two‑sample SPM t‑test | Asymmetry waveform |
| All presentations | Discrete variables | Within‑presentation bilateral differences in discrete outcomes | Paired t‑tests (0D) | Both limbs |
| All presentations | Discrete variables | Between‑presentation differences in discrete bilateral asymmetry | Independent t‑tests (0D) | Bilateral asymmetry |
